# Communicative and linguistic factors influencing language development at 30 months of age in preterm and full-term children: a longitudinal study using the CDI

**DOI:** 10.3389/fpsyg.2023.1177161

**Published:** 2023-07-24

**Authors:** Anastasiia Ogneva, Miguel Pérez-Pereira

**Affiliations:** Department of Developmental and Educational Psychology, University of Santiago de Compostela, Santiago de Compostela, Spain

**Keywords:** low-risk preterm children, gestures, lexical development, grammatical development, determinants of language development

## Abstract

**Introduction:**

Previous studies showed that very preterm children have a delay in communicative (gestures) and linguistic development as compared to full-term children. Earlier use of gestures, as well as of word comprehension and production, have been found to be predictive of subsequent word production and/or language delay in both very preterm and full-term children. Not many studies on communicative antecedents of language, however, have been carried out with low-risk preterm children in comparison to full-term children.

**Methods:**

In the present study a sample (*N* = 142) of low-risk preterm children has been followed using the Galician version of the Communicative Development Inventories (CDI) at the ages of 10, 22, and 30 months of age and their results were compared to the results from a sample (*N* = 49) of full-term children at the same ages. The determinants of language measures (vocabulary and grammar) at 30 months of age have been studied through linear regression analyses.

**Results:**

ANOVA results indicate that there were no significant differences between the groups in any of the measures obtained with the CDI at any time, nor were there any differences in lexical or grammatical developmental trajectories between both groups (repeated measures ANOVA). Linear regression analyses showed that the predictors of language at 30 months of age are somewhat different for the full-term than for the preterm group.

**Discussion:**

While the use of first communicative gestures at 10 months is a predictor of word production at 30 months of age for the full-term group, participation in games and routines seems to play a significant predictive role for preterm children. Word production at 22 months is the factor with a major incidence on word production at the age of 30 months for both groups. Previous specific measures of grammatical development have a clear determinant role in grammar measures at 30 months of age for the full-term children, while in the case of preterm children previous lexical development seems to be more relevant.

## 1. Introduction

### 1.1. Early precursors of language in full-term children

Children start to use their first words around 12 months of age. They show other abilities shortly before that time, however, which are considered to be precursors of language. Among these are the abilities to imitate the actions of the caregiver, to participate in social games and daily routines, and to use gestures. From a theoretical point of view, the emergence of these abilities has been linked to important advances in socio-cognitive capacities. From Vygotsky’s (1978) pioneering proposal that the use of gestures arises as a result of the interiorization process that is forged in social interactions, other authors have developed this idea, and have proposed other socio-cognitive abilities to explain the emergence of imitation of adults’ actions, participation in interaction routines and social games, and use of gestures, as well as the first communicative actions (protoimperatives and protodeclaratives; Bates et al., 1975; Ratner and Bruner, 1978; Bruner, 1983; Nelson, 1985; Tomasello et al., 1993; Tomasello, 2003). Those proposals share a socio-pragmatic perspective on language acquisition, which is inspired in authors such as Wittgenstein (Nelson, 2009). According to Tomasello and others (Tomasello et al., 1993; Carpenter et al., 1998; Tomasello, 2003), the attainment of three socio-cognitive abilities is the foundation not only for the development of communicative abilities but also for the development of first language: (1) joint attention, (2) intentional reading, and (3) cultural learning or the capacity of role reversal imitation. These three abilities emerge between 9 and 12 months of age in this order and are also crucial for shared intentionality (Tomasello and Carpenter, 2007; Tomasello, 2008).

Gestures and their role for the development of language have been more widely studied than imitation of actions or participation in social games and daily interactional routines.

Young children communicate using gestures before they produce their first words (Bates, 1976). Gestures are reported to reflect cognitive and socio-cognitive developmental changes in infancy (Tomasello, 2003; Kuvač et al., 2014). There are several systems of gesture classification. According to Farkas (2007), most current studies follow the classification by Capirci et al. (1996) who described deictic and symbolic gestures which are the earliest to appear in child communication. Iconic gestures, proposed by Nicoladis et al. (1999), are produced when children have already acquired some verbal language.

At around 9–10 months, children start using deictic gestures which can be considered the first signs of intentional communication and their referential meaning is given entirely by the context (Özçalişkan et al., 2014). The most commonly studied deictic gestures are pointing, reaching, showing and giving (Crais et al., 2004). Children use them to draw parent’s attention to an object, for example, pointing at a bottle to indicate a bottle. Deictic gestures constitute a useful tool for children to refer to objects before they can verbally name them. Previous research suggests that pointing at a particular object increases the chances for the child to learn the word for an object (Iverson and Goldin-Meadow, 2005) and generally paves the way for verbal language development (Goodwyn et al., 2000). A second class of gestures, referred to as “symbolic” or “representational,” typically appear in children’s communication at approximately 12–15 months of age. Some authors distinguish between social gestures, action-related or object-related gestures (Farkas, 2007; Stefanini et al., 2009). Gestural routines are part of everyday interactional routines (e.g., waving “bye-bye” or shaking head for “no”) and are also considered to be the first communicative gestures (Fenson et al., 1993). Action-related gestures and object-related gestures are used to refer to the function of a referent or the referent itself.

The early gestures produced by children are not only considered to be precursors of words, but they are also predictors of them. Previous research has shown that it is possible to predict a large portion of the words that will eventually appear in children’s spoken vocabulary. Lexical items that were initially expressed with gestures appear in the verbal lexicon 3 months later (Rowe et al., 2008). Silva et al. (2017) studied communicative development of Portuguese infants aged between 8 and 15 months and concluded that although gestures are a good predictor of vocabulary development, they are more closely associated with vocabulary comprehension than with vocabulary production. Similarly, Cadime et al. (2017), who studied 48 children at 9, 12 and 15 months of age longitudinally with the Portuguese version of the Communicative Development Inventories (CDI), found that the total number of actions and gestures and the number of early gestures produced at 9 and 12 months predicted the number of words comprehended at 15 months of age. The number of words produced, however, was predicted by actions and words only at 9 and 12 months, but not later.

Rowe and Goldin-Meadow (2009) suggested that early gestures predict later language development in a selective manner. It was found that gesture use at 18 months selectively predicted lexical and syntactic skills at 42 months. Specifically, different meanings conveyed in gestures at 18 months predicted vocabulary at 42 months, but the number of gesture and speech combinations did not predict later vocabulary. Similar results were obtained by Kuvač et al. (2014) who carried out a study with 250 infants aged 8–16 months to analyze predictive roles of different types of gestures on the onset of first word categories in early expressive vocabulary. According to their results, different types of gestures predict different types of words. For example, open-class words (such as common noun and predicates) were strongly predicted by object gestures, whereas social terms were predicted by gestural routines.

Some studies have reported an association between earlier and later verbal abilities in typically developing children. Specifically, early comprehension is claimed to be associated with later receptive (Bates et al., 1988) and expressive vocabulary (Bavin et al., 2008). The association between production of words and gestures and later expressive vocabulary skills has been reported as well (Capirci et al., 1996). Some studies also have suggested that typically developing children benefit from observing referential iconic gestures in narrative comprehension (Dargue and Sweller, 2020) and that increasing exposure to gestures produced by mothers may impact 10–12 month old infants’ language development through an effect on sensorimotor brain activity (Salo et al., 2023).

Gestures have also been found to be correlated with language impairment in some studies. More specifically, children at later risk of language impairment were found to present significantly less gesture use and vocabulary abilities compared to the typically developing peers. Therefore, scarce gesture use may potentially serve as a diagnostic tool to identify children at risk for language impairment (Jackson-Maldonado, 2004; Goldin-Meadow et al., 2014; Hsu and Iyer, 2016). Similarly, Thal et al. (1991) conducted a follow-up study with 10 children who were 10% below their age peers in verbal language production when first measured and it was found that those children presented a significantly lower use of gestures.

The participation of infants in social games appears for the first time even before 8 months of age, and it becomes a very frequent activity in infants’ lives. Children’s participation in everyday routines and first social games, such as peek a boo, is firstly scaffolded by the adult, although later the children will be able to initiate social games by themselves. In this way they can affect the behavior of their parents and convey their wishes to play (Clark, 1978; Ratner and Bruner, 1978; Bruner, 1983; Camaioni and Laicardi, 1985). Participation in conventional social games is considered to favor language development because of the characteristics of social games: their high predictability, which will allow the children to anticipate what will happen next, and their organization in participants’ turns, which may be reversible (Ratner and Bruner, 1978; Bruner, 1983). When the integrated multimodal structure of the game is violated, children are less engaged in it (Fantasia et al., 2014).

Infants’ first words are mainly produced in contexts of social games, in which the mothers tend to use very repetitive and predictable language (Bruner, 1983; Camaioni and Laicardi, 1985). This finding has been corroborated by Dromi and Zaidman-Zait (2011) who found that participation in social games (peek a boo) and book reading activities (but not use of pointing gestures) was significantly associated with the number of words produced by 154 children between 12 and 15 months of age. The authors conclude that the transition into conventional language takes place within a rich context of non-verbal communicative behaviors (Dromi and Zaidman-Zait, 2011).

As for role reversal imitation of adult actions, its appearance is closely linked to the emergence of cultural learning, which is based on previous abilities for shared attention and the interpretation of the other’s intention (intentional reading). Role reversal imitation is of capital importance for the learning of cultural tools (spoons, glasses, keys, computers …) and the appropriation of culture by human beings (Tomasello et al., 1993). Manifestations of cultural learning capacity appear around 10–12 months of age. After this age, children learn to use many things relating to their cultural background and how to behave in different circumstances. Progress in role reversal imitation ability occurs after 12 months of age. Carpenter et al. (2005) found that imitations of other’s actions are just as common in typically developing infants at 12 months of age as at 18 months; role reversal actions which involve acting on an object (triadic object related role reversals), however, are more difficult for 12 month old children than for those of 18 months. Children with autism spectrum disorder were found to have a very limited use of role reversal imitation (Carpenter et al., 2005). The authors found positive relations between role reversal imitation and measures of language development at 18 months of age. Imitative actions, language comprehension, and language production at 18 months uniquely contributed to the prediction of late development of language production at 30 months in a sample of nearly 30,000 Norwegian children, while pointing gestures did not (Zambrana et al., 2013). Action imitation, therefore, seems to be a better predictor of late language development than pointing gesture.

### 1.2. Early precursors of language in preterm children

Although there have been several studies that have examined the development of gestural communication among atypically developing children such as children with Down syndrome (Iverson et al., 2003) or children with Williams syndrome (Laing et al., 2002), research focused on preterm children is still rare. Preterm birth has been reported to be a factor that negatively affects early communication development (during the period of 8–15 months), especially among those children who were born under 32 weeks of gestation (Pérez-Pereira et al., 2014).

Suttora and Salerni’s longitudinal observational study (Suttora and Salerni, 2012) explored the development of communicative gestures in 16 preterm children [mean gestational age (GA) = 30 weeks] and two groups of full-term children at different periods (12, 18, and 24 months of age). Deictic gestures were the most frequently produced by the FT and the PT children at 12, 18, and 28 months of age, followed by referential gestures. No differences in the use of gestures or gesture types were found between the FT and the PT children. Their findings suggest that for preterm children the production of communicative pointing at 12 months is positively related to the linguistic skills at 18 and 24 months of age. The presence of pointing in children’s communication at 12 months predicted their vocabulary size at 18 months and the spontaneous lexical productivity and complexity at 24 months.

Sansavini et al. (2011b) reported that 104 preterm children (mean GA = 29.5 weeks), who were measured through the Italian short form of the CDI, showed a slower rate of development in gesture/action production, word comprehension, and word production than 20 FT children, with an increasing divergence between the two groups from 12 to 24 months. Nevertheless, the preterm sample used in these studies included very or extremely preterm children (<32 and <28 weeks of gestation) or children with very low birth weight (<1,500 g). Lexical competencies at 12 months, together with gestures/actions at 18 months, were predictive of word production at 24 months.

There is controversy on the long-term effect of early development of gestures and receptive and expressive language on later language skills. The results of Pérez-Pereira et al.’s (2014) study indicate that although gestures and early word comprehension (measured at 10 months) predict very early word production, this effect disappears with time showing no correlation after 24 months of age. Similar results were obtained by Stolt et al. (2014) who found a significant effect of gestures measured at 15 months on language scores at 24 months of age, but no significant predictive value of gestures measured at 9 months on language at 24 months. However, Stolt et al. (2016) reported that the development of gestures measured between the ages of 9 months and 15 months, as well as the receptive and expressive language ability measured at 24 months, correlate significantly and positively with language skills at 60 months in preterm children with very low birthweight (GA range = 23–34 weeks of gestation).

There is controversy as well in the results of research focused on language development in preterm children. On one hand, many studies have reported that preterm (mainly very and extremely preterm) children present smaller vocabulary size as well as lower grammatical skills in comparison with full-term children (Sansavini et al., 2010, 2011a; Stolt et al., 2012, 2013; Varela-Moraga et al., 2023).

On the contrary, a few studies conducted with healthy preterm children with a wider range of gestational age have not found differences between full-term and preterm children in language acquisition (Sansavini et al., 2006; Gayraud and Kern, 2007; Pérez-Pereira et al., 2014; Pérez-Pereira and Cruz, 2018; Suttora et al., 2020). Sansavini et al. (2006) investigated early lexical and grammatical development in Italian preterms (GA < 33 weeks) and fullterms at the age of 30 months. The result of this study suggested that most of the preterm sample displayed linguistic abilities within the normal range. As for the factors influencing preterms’ language development, birthweight, gestational age and gender were shown to have the major effect. Specifically, total words number and MLU scores are affected by an extremely low birthweight, a gestational age <31 weeks and male gender.

Gayraud and Kern (2007) studied early grammatical and lexical development in 323 preterm children compared to full-term peers at 24 months using the French MacArthur-Bates parental report. Preterm children were grouped according to their GA: extremely preterm (under 28 weeks of gestation), very preterm (between 28 and 31 weeks of gestation) and moderately preterm (between 32 and 36 weeks of gestation). Results showed that preterm children understood fewer words and produced more games, routines and onomatopoeia words. Overall, no differences were found between preterm and full-term children, if the extremely preterm group was not considered. The results obtained in this study showed that pre-term children obtained scores similar to those of younger full-term children. Therefore, the authors suggest that differences observed between groups are delays rather than deviances from the typical course of language development. These authors suggest that, as preterm children mature, differences between preterm and full-term children decline (Gayraud and Kern, 2007).

In Pérez-Pereira et al.’s (2014) study no significant differences were found between 3 groups of preterm children with different GAs (extremely and very preterm, moderately preterm and late preterm) and full-term children in communicative, lexical or grammatical development.

Preterm and full-term children were also reported to have similar developmental paths in lexical development. Specifically, Pérez-Pereira and Cruz (2018) compared the vocabulary size and composition of preterm children with different gestational age (very and extremely preterm group: 26–31 weeks, moderately preterm group: 32–33 weeks, late preterm group: 34–36 weeks) and full-term children at different periods of time (10, 22, and 30 months). Growth curve analyses showed no differences in word categories or vocabulary size among the four groups of participants. The main predictors of total vocabulary and word categories at 30 months were cognitive scores and word production measured at 22 months.

To the best of our knowledge, no study has been performed with PT children to investigate the effect of action imitation or participation in social games on later language development.

The existence of parental inventories has made it possible to gather an extensive amount of information on early communicative and linguistic development. The CDI permits the assessment of: use of gestures, participation in social games and routines, action imitation ability, as well as word comprehension and production between 8 and 15 months of age. The CDI also enables us to explore the abilities of children in word production, as well as morphosyntactic development (see the instruments section below) between 16 and 30 months of age. Therefore, the CDI seems to be an adequate, reliable and easy to use instrument to explore longitudinal relationships between early communicative and linguistic abilities and later language development.

To summarize, communicative antecedents of language have not been studied to a great extent in low-risk preterm children, children without associated medical complications. Therefore, the main aims of this study are the following:

1. To compare (cross-sectional analysis) the results obtained by the PT and the FT groups in the measures taken at 10, 22, and 30 months of age (see the instruments section and the analysis performed section below).
2. To compare the developmental trajectories throughout time of preterm and full-term children in the measures taken at different occasions (see the analysis performed section below).
3. To identify the factors predicting language development (word production, use of regular morphemes, MLU3 and sentence complexity) at 30 months of age in preterm and full-term children (see the analysis performed section).

The hypotheses of the study are as follows:

1. There will not be significant differences between the preterm and full-term groups of children in the scores obtained in the different measures of the Inventario para o Desenvolvemento de Habilidades Comunicativas: the Galician CDI (IDHC) taken at 10, 22, and 30 months of age, given the low-risk condition of the PT children.
2. No significant differences between the FT and the PT groups (inter-subjects differences) will exist in the developmental trajectories throughout time of the measures taken on different occasions: word production, MLU3, sentence complexity and regular suffixes.
3. The use of first communicative gestures will have an influence on some of the linguistic measures taken at 30 months of age in the FT as well as in the PT children (see the analysis performed section).
4. There will be variations in the determinants which have an effect of later language development (30 months of age) between the full term and the preterm children.

## 2. Methods

### 2.1. Participants

This study has been carried out using part of the data gathered in a long longitudinal project carried out with an initial sample of 150 low-risk preterm children (PT) and 49 full-term (FT) children who were studied from birth until their 9th birthday. The children and their families were recruited from 4 different hospitals in Galicia (Spain).

For the purposes of the present study, data on language and communicative development gathered at 10, 22, and 30 months of age will be presented. Corrected age has been used for the PT participants.

At 10 months of age the sample comprised 142 PT children, and 49 FT children. There were 45 PT children below 32 weeks of gestation, 36 PT children with a GA of 32 or 33 weeks, and 61 PT children with a GA between 34 and 36 weeks. The next assessment occasion took place when the children were 22 months of age. At this moment, there were 137 PT children, and 43 FT children. There were 43 PT children below 32 weeks of gestation, 36 PT children with a GA of 32 or 33 weeks, and 58 PT children with a GA between 34 and 36 weeks. At 30 months of age the children were assessed again. At this time, the PT sample consisted of 117 children, and the FT sample of 37 children. There were 37 PT children below 32 weeks of gestation, 32 PT children of 32 or 33 weeks of gestation, and 48 PT children with a GA between 34 and 36 weeks.

PT children with further serious complications were excluded from the study. Among the exclusion criteria were babies suffering from periventricular leukomalacia (PVL), intraventricular hemorrhage (IVH) greater than grade II, cerebral palsy (as diagnosed up until 9 months of age), hydrocephalus, encephalopathy, genetic malformations, chromosomal syndromes, metabolic syndromes associated to mental retardation, or important motor or sensorial impairments. Neonates with Apgar scores below 6 at 5 min were also excluded.

Descriptive data of the children at different occasions are shown in Table 1.

**Table 1:** Descriptive data of the sample.

	*N*	GA mean (SD)	GA range	Apgar	BW mean (SD)	Gender (male)	Maternal education
PT newborn	150	32.60 (2.46)	26–36	7.87 (1.43)	1727 (0.447)	52.10%	25.3%^a^
							39.3%^b^
							35.3%^c^
FT newborn	49	39.84 (1.44)	37–42	8.08 (1.25)	3,378 (0.414)	51.00%	38.8%^a^
							26.5%^b^
							34.7%^c^
PT 10 m	142	32.61 (2.40)	26–36	7.94 (1.33)	1718 (0.430)	52.10%	23.9%^a^
							40.1%^b^
							35,9%^c^
FT 10 m	49	39.84 (1.44)	37–42	8.08 (1.25)	3,378 (0.414)	51.00%	38.8%^a^
							26.5%^b^
							34.7%^c^
PT 22 m	137	32.62 (2.41)	26–36	7.94 (1.30)	1721 (0.435)	52.60%	24.8%^a^
							40.9%^b^
							34.3%^c^
FT 22 m	43	39.70 (1.48)	37–42	8.13 (1.20)	3,373 (0.433)	53.50%	39.5%^a^
							23.3%^b^
							37.2%^c^
PT 30 m	117	32.56 (2.49)	26–36	7.94 (1.27)	1712 (0.428)	56.50%	22.6%^a^
							45.2%^b^
							32.2%^c^
FT 30 m	37	39.76 (1.49)	37–42	8.16 (1.25)	3,377 (0.443)	51.40%	37.8%^a^
							27.0%^b^
							35.1%^c^

GA, Gestational age.

BW, Birth weight.

Maternal education:

a Basic education.

b High school and technical school education.

c University degree.

Both groups were similar in terms of distribution by gender [*X*^2^(1) = 0.025, *p* = 0.874], mothers’ education [*X*^2^(2) = 4.008, *p* = 0.135] and Apgar score [*t*(197) = −0.909, *p* = 0.365], at the beginning of the study, and throughout the duration of the study.

The former data (Table 1) indicate that the children who still continued in the project at 30 months of age had similar characteristics to the original sample. Thus, there was no substantial change in sample composition throughout time.

Taking into account the Apgar mean score, the inexistence of children with serious medical complications, and the characteristics of their families (mother’s education), the sample of PT children may be considered as a low-risk sample.

### 2.2. Instruments

The children participating in the study were assessed at 10, 22, and 30 months of age through the *Inventario para o Desenvolvemento de Habilidades Comunicativas* (IDHC; Pérez-Pereira and García Soto, 2003; Pérez-Pereira and Resches, 2011), a well-known parental inventory which is the Galician version of the MacArthur-Bates Communicative Development Inventories (CDI; Fenson et al., 2007). The form for children between 8 and 15 months (*Palabras e Xestos* “Words and Gestures”) of this parental inventory has been administered to the participants’ parents when the children were 10 months of age. This form evaluates different aspects of communicative abilities and first language (see Pérez-Pereira and García Soto, 2003; Pérez-Pereira and Resches, 2011; Pérez-Pereira, 2008) for a description of the instrument. From the results obtained, the following measurements have been considered for the analysis: Phrases (understanding of phrases), vocabulary comprehension, vocabulary production, first communicative gestures, games and routines, actions (total score obtained from the sum up of actions with objects, pretending to be a parent, imitating other adult actions).

The form *Palabras e Oracións* (Words and sentences) for children between 16 and 30 months of age was administered to the parents (mainly mothers) of the participants at 22 and 30 months of age. This form assesses different aspects of lexical and grammar development of children (for a description of the instrument see Pérez-Pereira and García Soto, 2003; Pérez-Pereira and Resches, 2011; Pérez-Pereira, 2008). The following measures were used for the analyses: Word production, Use of regular suffixes (forms of words), Mean length of the three longest utterances in words produced by the child (MLU3) and Sentence complexity.

In addition, a complete interview was applied to the mothers in order to get information on the sociodemographic characteristics of the families (educational level of both parents, occupation, family composition, etc.), the health of the children and the caregivers, and other relevant characteristics of the children.

### 2.3. Procedure

Parents’ consent, and approval (2008/010) by the Galician Ethics Committee of Clinical Research were obtained before the beginning of the research.

The interview to mothers was administered shortly after the birth of the children, and again at 30 months of age in order to update information.

The IDHC-words and sentences were administered to the parents of the participants when they were 10 months of age (+15 days), while the IDHC-words and sentences were applied when the children were 22 and 30 months of age (+15 days).

### 2.4. Analysis performed

ANOVA analyses have been performed to compare the results obtained by the FT and the PT groups in the different measures taken. The effects of the independent variable (PT vs. FT group) on the following dependent variables have been analysed: understanding of phrases, vocabulary comprehension, vocabulary production, first communicative gestures, games and routines and actions (obtained through the IDHC at 10 months of age); word production, use of regular suffixes, MLU3 and sentence complexity (obtained through the IDHC at 22 and 30 months of age). Previous analysis with the division of the PT children into three different GA groups (<32 weeks, 32–33 weeks, and 34–36 weeks) have not found any significant difference among them; for this reason, all PT children were integrated into a single group.

Repeated measures ANOVAs have been carried out with measures of word production taken at 10, 22, and 30 months of age and with measures of MLU3, sentence complexity and use of regular suffixes, at 22 and 30 months of age, in order to test whether developmental trajectories differed between the two groups (PT vs. FT) or not. Therefore, 2 different models were used: (1) a 2 (age) × 2 (group) repeated measures ANOVA has been used in the case of the measures of which there were two different scores obtained at 22 and 30 months of age: MLU3, sentence complexity and use of regular suffixes; (2) a 3 (age) × 2 (group) repeated measures ANOVA has been used to analyse the scores obtained in word production at 10, 22, and 30 months of age. In this way we could test if there were intra-subjects differences (age related differences in the same participants), inter subjects differences among groups (PT vs. FT), and a combined effect age × group.

Linear regression analyses have been performed to identify those determinants of language measures (dependent variables (DV)) taken at 30 months of age (word production, MLU3, sentence complexity and use of regular suffixes). Forward method has been used. The following measures have been introduced as independent variables. In Block 1 a series of measures taken at the age of 10 months were introduced:

Phrase understanding at 10 months of age.
Word comprehension at 10 months of age.
Word production at 10 months.
First communicative gestures at 10 months.
Games and routines at 10 months.
Total imitation at 10 months of age.
In Block 2, measures taken at 22 months of age were added:
Word production at 22 months of age.
Regular suffixes at 22 months.
MLU3 at 22 months of age.
Sentence complexity at 22 months.

The use of these two blocks allows us to identify the effect of variables taken at a longer distance (10 months of age), the effects of which could not be detected if they were mixed with more proximal variables in the same block.

## 3. Results

Table 2 shows descriptive data and ANOVA results.

**Table 2:** Scores of the language measures of the two groups and ANOVA results.

		GA group mean (SD)					
	N PT/FT	Preterm	Full-term	*F*	Degrees of freedom	Sign.	Partial eta squared
Phrases 10 m	142/49	13.67 (6.5)	14.45 (6.4)	0.523	190	0.470	0.036
Word underst. 10 m	142/49	79.17 (74.1)	71.86 (58.8)	0.391	190	0.533	0.034
Word product. 10 m	142/49	5.30 (7.7)	6.39 (21.9)	0.260	190	0.610	0.030
First gestures 10 m	142/49	7.09 (2.6)	7.53 (2.5)	1.035	190	0.310	0.044
Games and rout. 10 m	142/49	4.37 (1.8)	4.96 (1.6)	3.794	190	0.053	0.074
Total imitation 10 m	142/47	9.66 (6.4)	10.93 (7.5)	1.235	188	0.268	0.048
Word product. 22 m	137/43	158.65 (147.2)	173.77 (137.1)	0.356	179	0.552	0.035
Regular suffixes 22 m	137/43	1.53 (2.1)	1.79 (1.9)	0.506	179	0.478	0.038
MLU3 22 m	135/43	2.65 (2.1)	2.69 (2.0)	0.280	177	0.597	0.033
Sentence compl. 22 m	137/43	2.53 (4.9)	2.35 (4.3)	0.048	179	0.827	0.021
Word product. 30 m	117/37	416.19 (175.6)	411.49 (171.3)	0.020	153	0.887	0.019
Regular suffixes 30 m	112/35	5.86 (2.8)	5.20 (3.2)	1.343	146	0.248	0.062
MLU3 30 m	106/37	7.00 (4.4)	7.05 (5.7)	0.003	142	0.956	0.008
Sentence compl. 30 m	112/35	20.81 (14.3)	20.49 (13.3)	0.014	146	0.905	0.018

As can be observed, no significant difference between the PT and the FT groups is found in any of the measures. Only one trend is found (*p* = 0.053) in Games and routines. Size effects are very low, ranging from 0.008 (MLU3 at 30 months of age) to 0.074 (Games and routines at 10 months), which indicates that the effect of group (PT vs. FT) is minimal on the different measures of language and communicative development taken.

The results of the repeated measures ANOVAs indicate that there is a highly significant effect of age (intra-subjects differences) on Word production [*F*(2) = 309.430, *p* < 0.001, η^2^ = 0.805]; no significant combined effect of age x group is found [*F*(2) = 0.901, *p* = 0.408, η^2^ = 0.012]; no significant difference between groups (PT vs. FT) (inter-subjects effects) is found [*F*(1) = 0.145, *p* = 0.704, η^2^ = 0.001] in word production.

In relation to MLU3 as a dependent variable, the results of the repeated measures ANOVA indicate that there is a highly significant effect of age (intra-subjects differences) [*F*(1) = 136.055, *p* < 0.001, η^2^ = 0.496]; no significant combined effect of age × group is found [*F*(1) = 0.008, *p* = 0.928, η^2^ = 0.000]; no significant difference between groups (PT/FT) (inter-subjects effects) is found [*F*(1) = 0.070, *p* = 0.791, η^2^ = 0.001].

In relation to Sentence complexity as a dependent variable, the results of the repeated measures ANOVA indicate that there is a highly significant effect of age (intra-subjects differences) [*F*(1) = 208.618, *p* < 0.001, η^2^ = 0.592]; no significant combined effect of age × group is found [*F*(1) = 0.000, *p* = 0.985, η^2^ = 0.000]; no significant difference between groups (PT/FT) (inter-subjects effects) is found [*F*(1) = 0.010, *p* = 0.922, η^2^ = 0.000].

In relation to the Use of regular suffixes as a dependent variable, the results of the repeated measures ANOVA indicate that there is a highly significant effect of age (intra-subjects differences) [*F*(1) = 234.122, *p* < 0.001, η^2^ = 0.619]; no significant combined effect of age × group is found [*F*(1) = 0.2.505, *p* = 0.116, η^2^ = 0.017]; no significant difference between groups (PT/FT) (inter-subjects effects) is found [*F*(1) = 0.2.578, *p* = 0.592, η^2^ = 0.002].

The results of the longitudinal regression analyses with Word production at 30 months of age as a dependent variable for the preterm and the full-term groups appear in Tables 3, 4, respectively.

**Table 3:** Linear regression analysis: predictors of word production at 30 months of age: full-term group.

Predictors	Standardized β	Sig.	*R* ^2^	Change in *R*^2^	Change in *F*	Significance change in *F*	*F*	df	*p*
Model 1			0.174	0.174	6.966	0.013	6.966	1.33	0.013
First comm. gestures	0.417	0.013							
Model 2			0.275	0.101	4.436	0.043	6.064	2.32	0.006
First comm. gestures	0.347	0.031							
Word understand. 10 m	0.325	0.043							
Model 3			0.535	0.26	17.35	<0.001	11.891	3.31	<0.001
First comm. gestures	0.238	0.073							
Word understand. 10 m	0.219	0.097							
Word production 22 m	0.537	<0.001							

**Table 4:** Linear regression analysis: predictors of word production at 30 months of age: preterm group.

Predictors	Standardized β	Sig.	*R* ^2^	Change in *R*^2^	Change in *F*	Significance change in *F*	*F*	df	*p*
Model 1			0.057	0.057	6.767	0.011	6.767	1.112	0.011
Games and routines	0.239	0.011							
Model 2			0.093	0.036	4.382	0.039	5.677	2.111	0.004
Games and routines	0.327	0.001							
Word production 10 m	−0.209	0.039							
Model 3			0.383	0.29	51.746	<0.001	22.764	3.11	<0.001
Games and routines	0.156	0.072							
Word production 10 m	−0.229	0.007							
Word production 22 m	0.568	<0.001							
Model 4			0.41	0.027	4.93	0.028	18.915	4.109	<0.001
Games and routines	0.168	0.050							
Word production 10 m	0.194	0.021							
Word production 22 m	0.749	<0.001							
Regular suffixes 22 m	−0.254	0.028							

For the FT group, from the variables of Block 1 (taken at 10 months of age) First communicative gestures has been selected in Model 1 as having a significant effect on Word production (*p* < 0.05) and explains 17.4% of the variance (R2). In Model 2, First communicative gestures and Word understanding explain 27.5% of the variance (change in R2 increases 10.1% and reaches significance). Model 2 reaches significance (*p* < 0.01). When the variables of Block 2 are considered, the variance of the dependent variable explained is 53.5% and change in R2 increases 26% and reaches significance (*p* < 0.001). The two variables which are significant in Model 2 lose their significance in Model 3, and Word production at 22 months of age is the only variable which has a unique significant effect on word production at 30 months.

For the PT group, Model 1 incorporates Games and routines as a predictive variable of Word production at 30 months of age. The model reaches significance (*p* < 0.05) and explains 5.7% of the variance of the dependent variable. In Model 2 two variables, Games and routines and Word production at 10 months of age, have a significant effect. Model 2 explains 9.3% of the variance of the DV. Change in R2 increments 3.6% and reaches significance (*p* < 0.05). In Model 3 a new variable is included, Word production at 22 months of age, which reaches a high level of significance (standardized β). Now the variable Games and routines loses its significance, however Word production at 10 months continues to have a significant effect as well. The variance explained by Model 3 reaches to 38.3%, and change in R2 increases 29%, and is clearly significant (*p* < 0.001). Finally in Model 4 Use of regular suffixes is added to Games and routines, Word production at 10 months, and Word production at 22 months of age (all of which have a significant effect). Model 4 explains 41% of the variance of the DV and change in R2 reaches 2.7% and is significant (*p* < 0.05).

The results of the longitudinal regression analyses for the preterm and the full-term groups with MLU3 at 30 months of age as a dependent variable appear in Tables 5, 6, respectively.

**Table 5:** Linear regression analysis: predictors of MLU3 at 30 months of age: full-term group.

Predictors	Standardized β	Sig.	*R* ^2^	Change in *R*^2^	Change in *F*	Significance change in *F*	*F*	df	*p*
Model 1			0.26	0.26	11.601	0.002	11.601	1.33	0.002
MLU3 22 months	0.51	0.002							

**Table 6:** Linear regression analysis: predictors of MLU3 at 30 months of age: preterm group.

Predictors	Standardized β	Sig.	*R* ^2^	Change in *R*^2^	Change in F	Significance change in *F*	*F*	df	*p*
Model 1			0.564	0.564	130.421	<0.001	130.421	1.101	<0.001
MLU3 22 months	0.564	0.002							

In relation to the FT group, only MLU at 22 months appears as a predictor of MLU3 at 30 months of age in Model 1. The model reaches significance (*p* < 0.01), and the variance of the DV explained reaches 26%.

As for the PT group the results are similar. The only variable which appears to have effect on the DV is MLU3 measured at 22 months of age. Model 1 explains 56.4% of the variance and its significance level reaches *p* < 0.001.

The results of the longitudinal regression analyses for the preterm and the full-term groups with Sentence complexity at 30 months of age as a dependent variable appear in Tables 7, 8, respectively.

**Table 7:** Linear regression analysis: predictors of sentence complexity at 30 months of age: full-term group.

Predictors	Standardized β	Sig.	*R* ^2^	Change in *R*^2^	Change in *F*	Significance change in *F*	*F*	df	*p*
Model 1			0.196	0.196	7.566	0.01	7.566	1.31	0.01
Word production 10 m	0.443	0.01							
Model 2			0.311	0.115	5.017	0.033	6.782	2.3	0.004
Word production 10 m	0.38	0.02							
Games and routines	0.345	0.033							
Model 3			0.573	0.261	17.736	<0.001	12.956	3.29	<0.001
Word production 10 m	0.249	0.06							
Games and routines	0.348	0.009							
MLU3 22 months	0.528	<0.001							

**Table 8:** Linear regression analysis: predictors of sentence complexity at 30 months of age: preterm group.

Predictors	Standardized β	Sig.	*R* ^2^	Change in *R*^2^	Change in *F*	Significance change in *F*	*F*	df	*p*
Model 1			0.366	0.366	61.89	<0.001	61.89	1.107	<0.001
Word production 22 m	0.605	<0.001							

For the FT group, three models are obtained. In Model 1 Word production at 10 months has a significant effect (*p* = 0.01) and explains 19.6% of the variance of Sentence complexity at 30 months of age. In Model 2, a new variable, Games and routines, is added to Word production at 10 months. Model 2 explains a higher percentage of the variance of the DV (31.1%). The change in R2 reaches 11.5% and is significant (*p* < 0.05). Finally, in Model 3, Word production at 10 months loses significance (*p* = 0.06), Games and routines continues to have a significant effect and the incorporation of MLU3 at 22 months produces an increment of 26.1% in R2, a change which is clearly significant (*p* < 0.001). Model 3 explains 57.3% of the variance of sentence complexity at the age of 30 months and reaches a high level of significance (*p* < 0.001).

As for the PT group, the only variable which contributes to the explanation of the DV is word production at 22 months of age. In this case, Model 1 explains 36.6% of the variance of sentence complexity at the age of 30 months and the model reaches significance (*p* < 0.001).

The results of the longitudinal regression analyses for the preterm and the full-term groups with Use of regular suffixes as a dependent variable appear in Tables 9, 10, respectively.

**Table 9:** Linear regression analysis: predictors of regular suffixes at 30 months of age: full-term group.

Predictors	Standardized β	Sig.	*R* ^2^	Change in *R*^2^	Change in *F*	Significance change in *F*	*F*	df	*p*
Model 1			0.48	0.48	28.665	<0.001	28.665	1.31	<0.001
Regular suffixes 22 m	0.693	<0.001							
Model 2			0.572	0.092	6.418	0.017	20.047	2.3	<0.001
Regular suffixes 22 m	0.428	0.011							
MLU3 22 months	0.402	0.017							

**Table 10:** Linear regression analysis: predictors of regular suffixes at 30 months of age: preterm group.

Predictors	Standardized β	Sig.	*R* ^2^	Change in *R*^2^	Change in *F*	Significance change in *F*	*F*	df	*p*
Model 1			0.052	0.052	5.825	0.017	5.825	1.107	0.017
Games and routines	0.693	<0.001							
Model 2			0.303	0.252	38.321	<0.001	23.089	2.106	<0.001
Games and routines	0.047	0.583							
Word production 22 m	0.533	<0.001							

For the FT group, two models are obtained. In Model 1 Use of regular suffixes at 22 months of age explains 48% of the variance of the DV Use of regular suffixes at 30 months. Model 1 reaches significance (*p* < 0.001). In Model 2, a new variable is added to the former, MLU3 at 22 months of age. Now the variance explained reaches 57.2%, with an increment in R2 respect to Model 1 of 9.2%, which reaches significance (*p* < 0.05).

In relation to the PT group, 2 models are obtained. Model 1 contains Games and routines, which explains 5.2% of the variance and reaches significance (*p* < 0.05). In Model 2 a new variable is included, Word production at 22 months, and Games and routines loses significance. Model 2 explains 30.3% of the variance and has a significant effect on the use of regular suffixes at 30 months of age (*p* < 0.001). Change in R2 reaches 25.2% and is significant.

## 4. Discussion

In relation to objective 1, the results we found support hypothesis 1, which is that there will not be significant differences between the two groups in the scores obtained in the measures taken at any time. The results of the ANOVA are quite clear, and no significant differences were found between the PT and the FT groups, although the FT children show slightly higher results in all the measures taken except in word understanding at 10 months, sentence complexity at 22 months and in word production, regular suffixes and sentence complexity at 30 months of age. This indicates that the performance of the PT children seems to improve relatively as they grow older, supporting previous findings, because PT children obtain relatively better results when compared to FT children at 30 months of age (Gayraud and Kern, 2007; Pérez-Pereira, 2021). The fact that prematurity correction for age has been used may be behind these findings, since correction for age is less pertinent, and may have a higher effect, at 30 months of age than at 10 and 22 months of age. In addition, and coherently, size effects were minimal, and always below 0.075. The results found in terms of language development support the findings obtained in other studies carried out with low-risk preterm children (Sansavini et al., 2006; Gayraud and Kern, 2007; Pérez-Pereira et al., 2014; Suttora et al., 2020), and indicate that the results obtained in other studies with very and extremely preterm children, who were found to have smaller vocabulary size and grammatical skills than full-term children (Sansavini et al., 2010, 2011a; Stolt et al., 2012, 2013; Varela-Moraga et al., 2023), cannot be generalized to the overall group of preterm children.

The results found indicate that the precursors of language (use of gestures, participation in social games and routines, and role reversal imitation) are not delayed in the sample of low-risk PT children we studied. Suttora and Salerni (2012), using observational data, also found that there were no differences between preterm and full-term children in their use of communicative gestures at 12, 18, and 24 months of age. Our results are in contradiction with those obtained in another study (Sansavini et al., 2011b) which found a lower production of gestures/actions in the preterm children when compared to full-term children. This study, however, was carried out with very and extremely preterm children, and used the Italian CDI short form (not the complete form).

In relation to objective 2, the results we found indicate that the developmental trend that both groups follow are similar, supporting hypothesis 2. No significant differences were found in any of the repeated measures ANOVA performed on inter-subject differences (group effect) or combined effects of age by group. Therefore, the PT and the FT groups follow similar longitudinal trajectories both in lexical and in morphosyntactic development (MLU3, sentence complexity and use of regular suffixes), thus confirming hypothesis 2. These results agree with those found by Pérez-Pereira and Cruz (2018), who have found a similar pattern of lexical development in 3 groups of low-risk preterm children with different gestational ages and one group of full-term children through a growth curve analysis. On the contrary, the intra-subject effects found were important, which indicates an important change of the linguistic abilities (word production, MLU3, sentence complexity and use of regular suffixes) with age. The greatest intra-subject differences were found in word production (η^2^ = 0.805).

In relation to objective 3, the results found in the regression analyses do not seem to fully support hypothesis 3. Certainly, the use of first gestures has a predictive effect on word production at 30 months of age for the FT group, explaining over 17% of the variance, but not for the PT group. The results found give support to those found in other studies with FT children (Rowe et al., 2008; Rowe and Goldin-Meadow, 2009; Kuvač et al., 2014; Cadime et al., 2017; Silva et al., 2017). For FT children word understanding is also included as a predictive variable in model 2 and has a moderate effect on word production at 30 months. Other studies with FT children have also found an effect of word understanding on later word production (Capirci et al., 1996; Bavin et al., 2008; Zambrana et al., 2013). The effect of first communicative gestures on word production at 30 months of age in FT children appears as significant when the variables of Block 1 (taken at 10 months of age) are introduced as predictors, although this effect disappears when the variables of Block 2 are introduced. Then (Model 3 in Table 3), word production at 22 months of age is what has the predominant effect on word production at 30 months of age. This indicates that the effect of first communicative gestures fades as the age of the children increases, just as other studies have also found (Cadime et al., 2017). There is no other effect of first communicative gestures on any other grammatical measure at 30 months of age (MLU3, sentence complexity and use of regular suffixes). In this sense, the effect of the use of the first communicative gestures seems to be restricted only to lexical development in the case of full-term children.

In relation to the preterm children, the use of gestures has no significant effect on word production at 30 months of age, in contrast to what occurs for the low-risk FT group. This result disagrees with those of other studies carried out with very preterm or very low birth weight children which did find an effect of first communicative gestures on later word production (Sansavini et al., 2011a,b; Suttora and Salerni, 2012; Stolt et al., 2014, 2016). Other studies have also found that gestures are not significantly associated to word production in FT children (Dromi and Zaidman-Zait, 2011).

No effect of gestures on grammatical development was found either for the PT group, in contrast with the results found by other studies (Sansavini et al., 2011b; Stolt et al., 2014, 2016), which observed an effect of first communicative gestures on later grammatical development of very preterm children.

The results obtained for the low-risk PT group are considerably different and new. This time the use of first communicative gestures does not seem to have a significant effect on word production at 30 months of age, as already commented on; but the participation in games and routines, which has a modest (5.7%) although significant effect on the variance of word production at 30 months of age, does have a significant effect. To our knowledge this is the first time that this variable is reported as having a predictive effect on later language development of PT children and coincides with the findings of other studies carried out with FT children (Camaioni and Laicardi, 1985; Dromi and Zaidman-Zait, 2011). The characteristics of social games and routines, with their high predictability which will allow the children to anticipate what will happen next because of their regularity, the use of repetitive language by the mothers and their organization in participants’ turns (Bruner, 1983), all together seem to help children to understand and use first language, and surely constitute a very supportive environment. Therefore, the more children participate in social games and routines, the more first words they will use. Apparently, preterm children are particularly benefitted by the supportive context that social games and routines constitute.

In Model 2, word production at 10 months of age is also included as a predictor, this time with a negative relationship with word production at 30 months of age. In any case its effect seems to be very moderate. Finally, when variables measured at 22 months of age are included in Block 2, word production appears as the factor with the greatest influence on later vocabulary. The use of regular suffixes at 22 months is also included as having a modest (and negative) significant effect. Therefore, word production measured at 22 months has the largest influence on word production at 30 months of age for the PT group (similarly to the FT group). The effect of prelinguistic factors, however, still reaches a 0.50 significance level at 30 months of age (in the case of PT children), when variables taken at 22 months are included (see Model 4 in Table 4). This seems to point to a longer-lasting effect of variables taken at 10 months (participation in games and routines and word production at 10 months) on vocabulary development at 30 months of age in PT children (Rowe and Goldin-Meadow, 2009; Suttora and Salerni, 2012).

The reported effect of imitation on later word production (Carpenter et al., 2005; Zambrana et al., 2013) could not be confirmed in our study.

Therefore, hypothesis 3 is only partially confirmed, since first communicative gestures influenced later word production only in FT children, but not in PT children. In addition, this effect is circumscribed to word production, and no other grammatical measures have been affected by the use of first communicative gestures.

The regression analysis for the mean length of the three longest utterances at 22 months of age as DV shows similar results for the FT and the PT groups. In both cases the former measure of this same variable at 22 months is the only predictor which shows a significant effect. The amount of variance explained is 26 and 56.4% for the FT and the PT children, respectively. In this case a specific determinant effect of the same previous measure on a later measure of the same variable is observed. As far as we know, this relationship has not been previously noticed.

The other two grammatical measures obtained at 30 months of age taken as DVs seem to have different predictors for the FT and the PT groups, as hypothesized (hypothesis 4).

In relation to sentence complexity, word production and games and routines are the variables taken at 10 months of age which have a significant effect in the case of FT children. Their effect is noticeable (*R*^2^ = 0.311). When the variables measured at 22 months of age are included (Block 2), the variance explained increments of 0.261, and the effect of the three variables reaches 57.3%. Now (Model 3 in Table 7) the variables with a significant effect are MLU3 at 22 months of age and games and routines (word production at 10 months shows a trend). Again, the effect of games and routines lasts up to 30 months of age. In the case of the PT infants, no variable measured at 10 months of age seems to have an effect on sentence complexity, and the only variable which has a significant effect (*R*^2^ = 0.366) is word production at 22 months of age.

In relation to the predictors of the use of regular suffixes at 30 months of age, for the FT children no variable of Block 1 seems to have a significant effect, and the only two variables which reach significance are the use of regular suffixes at 22 months of age (Model 1), with an important effect (*R*^2^ = 0.480), and MLU3 at 22 months of age, with a modest effect (change in *R*^2^ = 0.092). For the PT children, games and routines has a modest, although significant, effect (*R*^2^ = 0.052) on regular suffixes at 30 months of age. When the variables of Block 2 are incorporated into the analysis, there is one variable, word production at 22 months of age, which shows a clear significant effect (change in *R*^2^ = 0.252), and games and routines loses significance (Model 2 in Table 10). The total variance explained reaches 30.3%.

Therefore, hypothesis 4 is confirmed, since the type of predictive variables which have a significant effect on word production, sentence complexity and use of regular suffixes at 30 months of age vary between the FT and the PT groups. The only exception occurs with MLU3, for which MLU3 at 22 months is the only explanatory variable found for the PT group as well as for the FT group. Its effect, however, is greater for the PT children.

One surprising and original finding is the role played by the participation in games and routines at 10 months of age as a predictor of later grammatical development for the FT and the PT children. In the case of PT children, this predicts the use of regular suffixes while in the case of FT children, its influence, which has a long-lasting effect, is on sentence complexity. Taking into consideration that participation in games and routines also has an influence on the vocabulary development of PT children, this variable stands out as a predictor of later language development. Probably, this is so because in the situations of social games and daily routines, social interaction between the child and the adult is promoted, and the possibilities of the child being exposed to language and using language increase, which will promote language development. Therefore, the higher the participation in games and routines, the better the development of language, as other authors have pointed out (Camaioni and Laicardi, 1985; Tomasello, 2003; Dromi and Zaidman-Zait, 2011; Kuvač et al., 2014; Hsu and Iyer, 2016).

Another difference between the predictors of some grammar development measures in PT and FT children is related to the degree of specificity of the factors. In the case of PT children, MLU3 at 22 months of age is the factor with the highest impact on sentence complexity at 30 months of age, and MLU3 together with regular suffixes at 22 months are the factors which have a significant predictive value for regular suffixes at 30 months of age. Meanwhile, for the low risk PT children, word production at 22 months of age is the only significant predictor of sentence complexity and it is the predictor (together with games and routines) with the highest impact on regular suffixes at 30 months of age. These results indicate that later grammatical development in FT children seems to be more dependent on specifically grammatical antecedents than in PT children, whose grammatical development is more dependent on previous lexical development, with the exception of the MLU3 already commented on.

The use of two blocks of predictive variables for the linear regression analyses allowed us to identify the effect of variables taken at a longer distance (10 months of age), the effects of which would be difficult to detect if they were mixed with more proximal variables in the same block.

## 5. Conclusion

The results found in the ANOVA analyses clearly indicate that low risk preterm children do not seem to have lower performance than full term children in any of the communicative and linguistic measures obtained at any time. In addition, the developmental trajectories of lexical and morphosyntactic abilities followed by the FT and the low-risk PT groups are similar.

There is a difference between the FT and the PT children in the type of predictive variables of later vocabulary production, sentence complexity and regular morphemes used at 30 months of age. Although the use of first communicative gestures does not have effect on later vocabulary development of the PT children, participation in games and social routines does seem to have an influence.

Later grammatical development of the FT ad the low risk PT children seems to be influenced by different previous linguistic abilities, which tend to be more specifically grammatical in the case of FT children.

Obviously, the use of parental reports limits the type of analysis which can be performed, since no information on the frequency of the behaviors studied is possible, and the information provided is prefixed by the instrument. The use of observational methodology would probably report more detailed information on parents-child interactions.

More specific analyses of the type of gestures and the type of words or morphemes used would be necessary to go more in depth into the relationships between preverbal communicative abilities and later language development.

Another limitation of the study is the reduced number of participants in the FT sample, which makes the analyzes less powerful.

## Data availability statement

The raw data supporting the conclusions of this article will be made available by the authors, without undue reservation.

## Ethics statement

The studies involving human participants were reviewed and approved by the Galician Ethics Committee of Clinical Research. Written informed consent to participate in this study was provided by the participants’ legal guardian/next of kin.

## Author contributions

MP-P was responsible for the conception of the study, data collection and analysis. AO and MP-P shared responsibility for drafting of the work and final approval of the version to be published.

## Funding

This research was funded by the Ministerio Economía Industria y Competitividad of the Spanish Government (Grants PSI2008-03905, PSI2011-23210, and PSI2015-66697-R to MP-P). Funds for open access publication fees were received from the Consellería de Educación, Universidade e Formación Profesional -Xunta de Galicia.

## Conflict of interest

The authors declare that the research was conducted in the absence of any commercial or financial relationships that could be construed as a potential conflict of interest.

## Editor’s note

Melita Kovacevic edited the article in collaboration with Maria-José Ezeizabarrena, University of the Basque Country UPV/EHU, Spain.

## Publisher’s note

All claims expressed in this article are solely those of the authors and do not necessarily represent those of their affiliated organizations, or those of the publisher, the editors and the reviewers. Any product that may be evaluated in this article, or claim that may be made by its manufacturer, is not guaranteed or endorsed by the publisher.
